# PaCHNOC: Packet and Circuit Hybrid Switching NoC for Real-Time Parallel Stream Signal Processing

**DOI:** 10.3390/mi15030304

**Published:** 2024-02-23

**Authors:** Peng Hao, Shengbing Zhang, Xinbing Zhou, Yi Man, Dake Liu

**Affiliations:** 1 School of Computer Science, Northwestern Polytechnical University, Xi’an 710072, China; peng.hao@mail.nwpu.edu.cn (P.H.); zhangsb@nwpu.edu.cn (S.Z.); 2 School of Information and Communication Engineering, Hainan University, Haikou 570228, China; dake@hainanu.edu.cn; 3 Ultichip Communication Technology, Beijing 100088, China; 4 School of Electronic Engineering, Beijing University of Posts and Telecommunications, Beijing 100876, China; manyi@bupt.edu.cn

**Keywords:** network on chip (NoC), hybrid NoC, real-time processing

## Abstract

Real-time heterogeneous parallel embedded digital signal processor (DSP) systems process multiple data streams in parallel in a stringent time interval. This type of system on chip (SoC) requires the network on chip (NoC) to establish multiple symbiotic parallel data transmission paths with ultra-low transmission latency in real time. Our early NoC research PCCNOC meets this need. The PCCNOC uses packet routing to establish and lock a transmission circuit, so that PCCNOC is perfectly suitable for ultra-low latency and high-bandwidth transmission of long data packets. However, a parallel multi-data stream DSP system also needs to transmit roughly the same number of short data packets for job configuration and job execution status reports. While transferring short data packets, the link establishment routing delay of short data packets becomes relatively obvious. Our further research, thus, introduced PaCHNOC, a hybrid NoC in which long data packets are transmitted through a circuit established and locked by routing, and short data packets are attached to the routing packet and the transmission is completed during the routing process, thus avoiding the PCCNOC setup delay. Simulation shows that PaCHNOC performs well in supporting real-time heterogeneous parallel embedded DSP systems and achieves overall latency reduction 65% compared with related works. Finally, we used PaCHNOC in the baseband subsystem of a real 5G base station, which proved that our research is the best NoC for baseband subsystem of 5G base stations, which reduce 31% comprehensive latency in comparison to related works.

## 1. Introduction

The heterogeneous microprocessors era has come [1]. Real-time parallel stream signal processing is a typical huge heterogeneous microprocessor application domain. Apart from the general computing, there are volume applications clarified as real-time parallel stream signal processing [2]. Their characteristics include: (1) Real-time requirement, which call for the system to get results in a limited run time; (2) Multi-task schedule, dispatch, and execution on parallel hardware; (3) Application specific and reasonably fixed yet predictable task flows; (4) A relatively stable data flow; (5) Using customized data precision to save the hardware cost and power consumption. A variety of applications, including high-end base band signal processing (base station and radar), parallel packet routing, high-end gaming, upmarket video codec, AI training, and AI inference, are applicable to this situation [3].

There are volume NoC platforms available. However, most them are packet switching NoCs for symmetric multiprocessing (SMP) architecture oriented for general computing [4]. This solution offers flexibility but with large routing latency and relatively high reorder buffer memory cost.

In a real-time parallel system for processing stream data, short data packets pop up frequently because the number of routing packets for task configuration is on the same magnitude comparing to the number of data packets [5]. Associated with a task execution, configuration packets comprise those for task configuration and task status reports.

**Figure 1:**
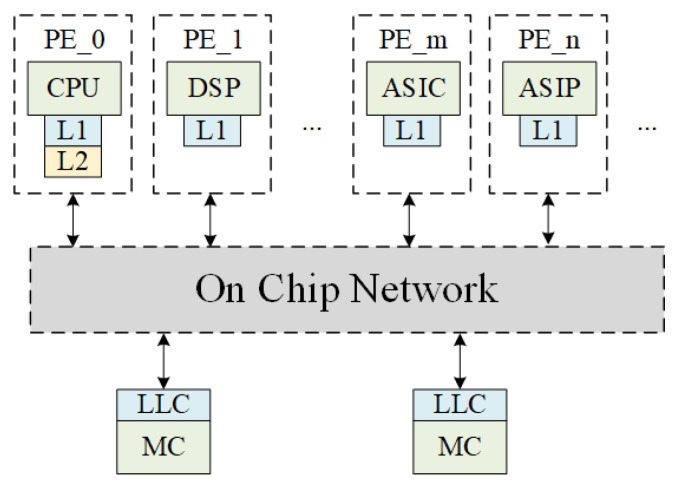
Example for an MPSoC. LLC stands for last level cache and MC stands for memory controller.

Hence, it is imperative to build a specialized NoC solution for multiprocessor system-on-chip (MPSoC) architectures that are designed to execute parallel and real-time stream signal processing tasks. Figure 1 illustrates an exemplary MPSoC platform, consisting of CPUs, DSPs, ASICs, and ASIPs (Application Specific Instruction-set Processor). We previously proposed a hybrid switching method PCCNOC [6] using packet-connected circuit. The PCCNOC proposed an approach presenting a temporally locked circuit, resulting in reduced latency and zero buffer cost. PCCNOC focused on packet-connected circuits, which exhibited outstanding circuit behavior. However, the efficiency for short data packet transmissions was found to be insufficient.

There exists literature on flexible packet switching NoC architectures. Packet switching offers high flexibility, while circuit switching provides excellent energy efficiency and short latency [7]. In the context of packet switching NoCs, the use of a pipeline for transfers at each router has been shown to enhance network utilization and to alleviate traffic congestion. Nevertheless, the implementation of packet switching necessitates significant energy consumption and additional space requirements for data storage and its reorder buffer in every router [8]. The energy efficiency of the NoC for SMP is diminished as a result of the substantial power consumption associated with data access in memory [9].

In contrast, circuit-switched NoCs set up and lock a complete channel from source to destination and transfer data with no intra-route data storage [10]. It achieves energy efficiency because data logic is minimized with only simple combinational logic of circuit-switched channel. Nevertheless, circuit-switched NoCs have high setup time because of the scheduling of channel setup and data transmission in serial. The extra setup cost can be negligible when the transferred data size is large. However, the size of a configuration vector for a task is usually small, so that the setup time is relatively large. In addition, statically configured circuit-switched networks are limited to fixed changing traffic permutations because of high channel setup latency [11]. In a Circuit-Switched NoC [12], allocated channel resources are prevented from processing other transfers, even if the circuit-switched channel is idle.

**Figure 2:**
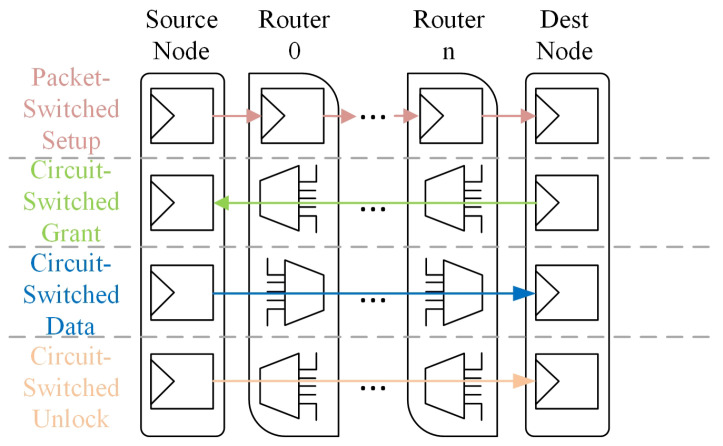
Hybrid switching transmission mechanism. The circuit setup for long data transmission.

Hybrid switching proposed achieves the advantages techniques on both circuit-switched low latency data transfer and packet-switched low channel setup cost [13]. In PCCNOC [6], packet-switched channel transfers circuit configuration vector, while circuit-switched channel transfers task data propagates on energy-efficiency and short latency due to buffer-free and no additional arbitration after locking the circuit. Circuit-switched channels release resources via unlocking the paths after completed transmission. Figure 2 shows the hybrid switching transmission mechanism for task data propagation. Generally speaking, it needs packet switch to set up a circuit switched channel. After receiving the grant signal, the data transmission is processed, and finally, its unlocking signal is generated to release the locked channel [7].

In this proposed study, we present a hybrid NoC architecture PaCHNOC for parallel and real-time stream signal processing. This architecture combines the benefits of packet switching and circuit switching. It reduces delays for both long data and short data. Moreover, the packet-connected circuit reduces the delay by temporally locked circuit, eliminating reorder buffer. Furthermore, the proposed approach, PaCHNOC, provides flex packet and data routing capabilities for efficient transmission of small-sized data, such as task setups and task running reports. PaCHNOC utilizes the packet switching method for when the size of the data packet is small (one packet can carry a complete configuration vector packet, so that the reordering buffer is not necessary). The primary areas of invention can be summarized as follows:

1. We have developed a revolutionary NoC architecture that integrates the advantages of packet switching and circuit switching, achieving short latency and low power for transferring both long data in circuits and short data in packets with high bandwidth and sufficient flexibility.
2. The Network Interface (NI) is designed with the purpose of automatically classifying packet types and employing specialized two transmission methods for transactions, hence enhancing overall network efficiency.
3. Router requester could be data sender or date receiver. Thus, it provides bus utilization and reduces system connection overhead.

The overhead of the system is rather minimal due to its buffer-free nature, which sets it apart from the baseline packet switching NoC. In contrast to classical hybrid NoCs [14], our technology employs multiplexing of the same lane for the transmission of both control signals and data.

The structure of the paper is as follows: Section 2 provides a concise overview of the relevant literature and previous research in the field; Section 3 provides an introduction to the reason and principle underlying the PaCHNOC; Section 4 examines the proposed NoC architecture and associated workflow; Section 5 examines the simulation environment and presents the outcomes of the suggested router architecture, as well as the synthesis result; and Section 6 draws a conclusive statement.

## 2. Related Works

Since its introduction by Dally [15], the concept of NoC has garnered significant attention and effort from both industry and academia, resulting in substantial advancements in its development. There exist multiple distinct branches in research and there exist various types of flow control mechanisms in communication systems, including message-based flow control, such as circuit switching, packet-based flow control, such as store and forward and virtual cut-through (VCT), and flit-based flow control mechanisms, such as wormhole and virtual channel [16].

**Figure 3:**
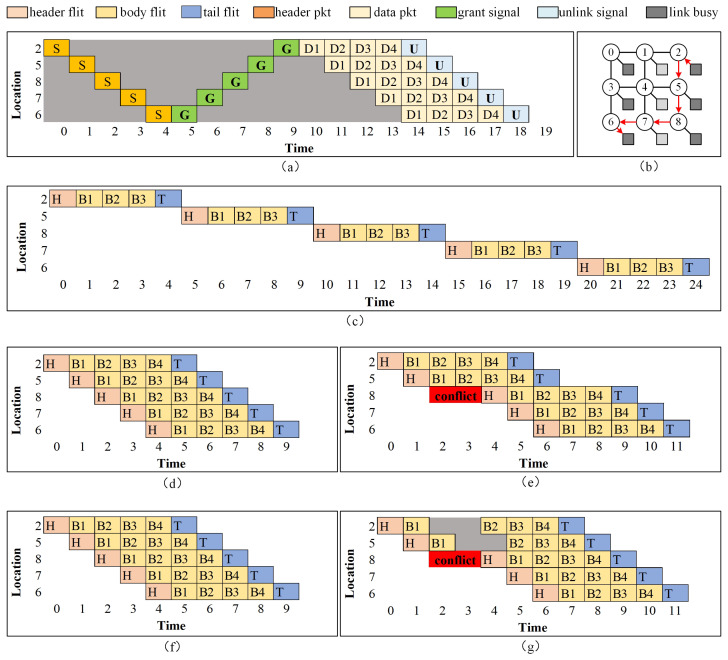
Pipelines for different switching method for a 3×3 NoC mesh using X-Y routing method. The data transmission task is from node 2 to node 6, as shown in (**b**). The subfigures are: (**a**) circuit switching flow; (**c**) store and forward data flow; (**d**) virtual cut through (VCT) work flow; (**e**) VCT with conflict at location 8; (**f**) wormhole (WH) data flow; (**g**) WH PS data flow with conflict at location 8.

The concept of message-based flow is the most basic and fundamental level of granularity in flow, as discussed in references [17,18]. Circuit switch is a typical method in telecommunications that establishes a dedicated communication path between two modules for the duration of a connection. Prior to transmitting the message, it is necessary to establish the whole route and allocate all communication channels from the source to the destination by employing a setup probe. After the setup process is completed, data is transmitted over the circuit channels. The utilization of buffers at routers is unnecessary due to the in-order data and the absence of contention. The circuit is dismantled once the transmission is complete. Figure 3a shows the pipeline for conventional circuit-switching NoC. The circuit establishing time occupied a high percentage, while data size is relatively small.

Packet-based flow control is a form of flow control that operates at an intermediate level of granularity. In the store and forward method, the allocation of links and buffers is performed on a per-packet basis. The head of the packet remains at the router until the full packet is received, after which it is forwarded to the next hop [19]. In the context of virtual cut-through, it is possible for flits to advance to the next hop prior to the current router receiving the tail flit. The pipeline for packet-based flow control can be glanced from Figure 3c, and VCT transmission flow is depicted in Figure 3d.

Fine-grained flow control refers to the regulation of data flow at a very detailed level, often referred to as “flits-level flow”. In contrast to VCT, the processing of flit in wormhole can commence once there is an adequate amount of buffering available for that specific flit. Routers are not required to possess buffers that are sized to accommodate packets, as this feature aids routers in adhering to stringent limitations on area and power consumption. Wormhole NoC is more efficient for buffer utilization with low latency, but suffers from head of line (HOL) blocking. The virtual channel architecture incorporates the utilization of numerous flit queues for each input port. Multiple datasets are transmitted across a common physical connection. This technology enhances the use of links and mitigates the occurrence of deadlock and HOL blocking. However, it alternates between fragments of various packets. Data serialization is a necessary requirement at the destination node. Figure 3f shows Wormhole data flow and Figure 3g shows data flow with congestion.

MRBS [20] manages all input buffers in a router as a register file style instead of dedicating a single buffer per input port, which efficiently utilizes the buffer space, especially for multicast traffic patterns. MRCN [21] uses destination router partitioning and traffic-aware adaptive branching technique to reduce packet routing hops and disperse channel traffic. NEBB (Non-Empty Buffer Bypass) [22] uses a mechanism that allows to bypass flits even if the buffers to bypass are not empty, which applies to wormhole and virtual cut-through to minimize latency and power. SBTR [23] introduces a cost-effective adaptive routing techniques that can forward long distance packets through specialized channels made of Transmission Line (TL). These extra TLs introduced in the chip reduce the diameter of the network, thereby reducing average packet latency.

Circuit switching is a highly effective strategy for minimizing on-chip communication delay in comparison to packet switching. This is due to the fact that packets can leverage pre-established circuits, hence bypassing the need for buffering, routing, hop-by-hop flow management, and arbitration at each individual hop. Nevertheless, the conventional circuit switching method is plagued by significant delays in establishing circuits and inefficient utilization of available bandwidth when transferred data size is very small, as shown in Figure 3a. The integration of circuit switching into a baseline packet-switched NoC in order to use the advantages of both switching mechanisms has been explored in many previous studies [18], namely hybrid NoC. In order to optimize the utilization of circuit switching while mitigating the impact of lengthy setup time, proactive circuit switching employs the strategy of concealing circuit setup latency through the pre-allocation of circuits. The system utilizes a control plane that transmits data request packets at increased voltage and frequency, resulting in enhanced speed. Additionally, a low-power data plane is employed to manage data packets.

CIMA [24] enhances a fundamental packet-switched router by incorporating a circuit-switching mechanism based on prediction. This mechanism deviates from conventional circuit-switching methods by effectively eliminating the overhead associated with circuit establishment. It reduces the setup delay by pre-allocating circuits for the precise duration required to transfer packets. NRR [25] presents a proposal and implementation of a multi-mode full reconfigurable router that supports a hybrid packet-switching architecture, which is dynamically reconfigurable to exchange between wormhole and virtual cut-through switching schemes at run-time. NRR is equipped a Quality-of-Services-driven arbiter to enhance the average performance for the best-effort service by using network resources efficiently. Besides, it is enhanced with the dynamically deadline-aware rerouting mechanism to reduce the waiting interval of the blocked packets.

## 3. Methodology

### 3.1. Motivation of the Proposed Solution

In the context of parallel real-time stream processing, the primary considerations of utmost significance is latency. In order to identify a more effective approach to meet the aforementioned requirements, it is necessary to carefully analyze all relevant factors and weigh them against one another. Network latency is calculated as Equation (1) [16]; its parameter description is listed in Table 1:

(1) $$T_{N}=t_{r}\times H+t_{w}\times \left(H-1\right)+T_{c}+T_{s}$$

In order to minimize the average network latency, researchers endeavor to decrease the magnitude of relevant variables. Within this set of parameters, the variables $t_{r}$, $t_{w}$, and $T_{s}$ are considered static, while *H* and $T_{c}$ are regarded as dynamic.

**Figure 4:**
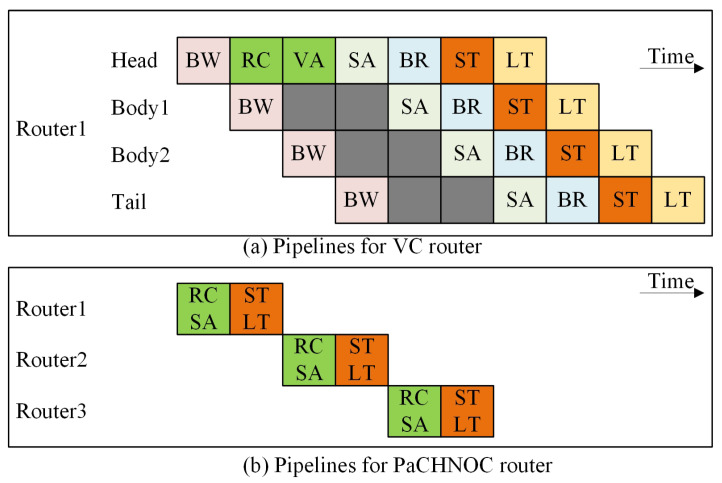
Router pipelines for packet switching NoCs. BW is short for buffer write, RC for reserve channel, VA for VC allocation, SA for switch allocation, BR for buffer read, ST for switch traversal, and LT for link traversal. (**a**) For the VC router [29]. (**b**) For the PaCHNOC router.

Figure 4a shows the traditional router pipeline diagram for packet switching NoCs. Some studies have tried to reduce $t_{r}$. Looking ahead combines buffer write (BW) and reserve channel (RC) in parallel to reduce routing latency. Smart [26] and PlugSmart [27] remove the dependence of latency on hops to reduce *H*. Wormhole [28] tries to avoid path conflict, so that the contention issue is properly handled.

Hybrid NoCs attempt to reduce transmission time cost by the following two ways [30]. First, to rely on the established route path on NoC, the *H* is, thus, significantly decreased. Second, since the established circuit is dedicated for the specific transaction, $T_{c}$ is zero when the route path is locked. In our proposed method, the router is bufferless so that it eliminates the time for writing and reading buffers (BW and BR).

For an ideal NoC transmission, where $t_{w}$ is 0 and there is no need for flit serialization and there is no congestion on the route, we could simplify the equation to Equation (2):

(2) $$T_{N}=t_{r}\times \;H+\;T_{c}$$

For baseline hybrid NoC transmission, and for a single transmission, the number of flits is *F*. Thus, $T_{hA}$ and $T_{pA}$ could be calculated by Equation (3) and Equation (4), respectively:

(3) $$T_{hA}=\frac{\left(t_{r}\times H+T_{c}+\;t_{c}\;\times F\;+\;1\right)}{F}=\frac{t_{r}\;\times \;H\;+\;1\;+\;T_{c}}{F}+t_{c}\;$$

(4) $$T_{pA}=\frac{{(t}_{r}\times H)\times F\;+T_{c}\;\;}{F}=\;t_{r}\times \;H+\frac{T_{c}}{F}\;$$

Furthermore, occupied link number is calculated by Equations (5) and (6):

(5) $$N_{hO}=\left(H+1\right)\times \frac{H}{2}+H\times \;F+\;T_{c}\times \;t_{oa}$$

(6) $$N_{pO}=H\times \;F+T_{c}$$

It is obvious that $N_{hO}$ is larger than $N_{pO}$. Hybrid transmission protocol would exacerbate congestion on NoC. According to Equations (3) and (4), the average latency for hybrid switching is smaller than that of packet switching when F>1, though occupied link numbers could be increased by increasing $T_{c}$. In our method, we try to combine the advantages for packet switching and circuit switching. When F=1, we use packet switching, and we use hybrid switching otherwise.

### 3.2. The 2D Mesh Topology

**Figure 5:**
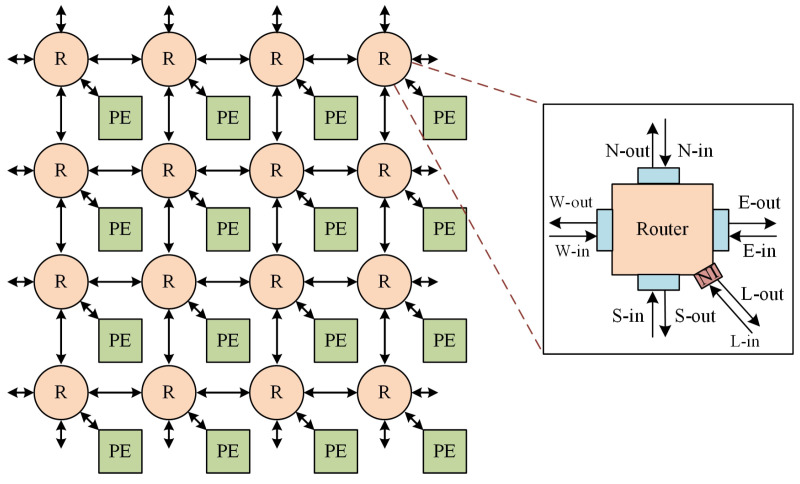
Baseline 4×4 NoC mesh.

The architecture of the PaCHNOC is based on a two-dimensional mesh topology. The composition of the system includes routers, network interfaces (NI), and provider edge (PE) devices. The design presented in Figure 5 illustrates a mesh structure with dimensions of 4×4. The router and network interface of the PaCHNOC have been changed from PCCNOC in order to accommodate the implementation of a novel transmission mechanism. The PaCHNOC has the potential to be configured as a K-ary 2-cube.

A router performs two primary functions: (1) establishing transmission circuits through the utilization of routing protocols, which enables the circuit switch of data, and (2) facilitating the transmission of small-sized data into one flit through conventional packet switching methods. The network router is equipped with five directional inputs and outputs: East (Ein), South (Sin), West (Win), North (Nin), and a local module (Lin). Similarly, it has five directional outputs: East (Eout), South (Sout), West (Wout), North (Nout), and a local module (Lout), as depicted in Figure 5.

### 3.3. Mixed Routing Protocol and Algorithm

**Figure 6:**
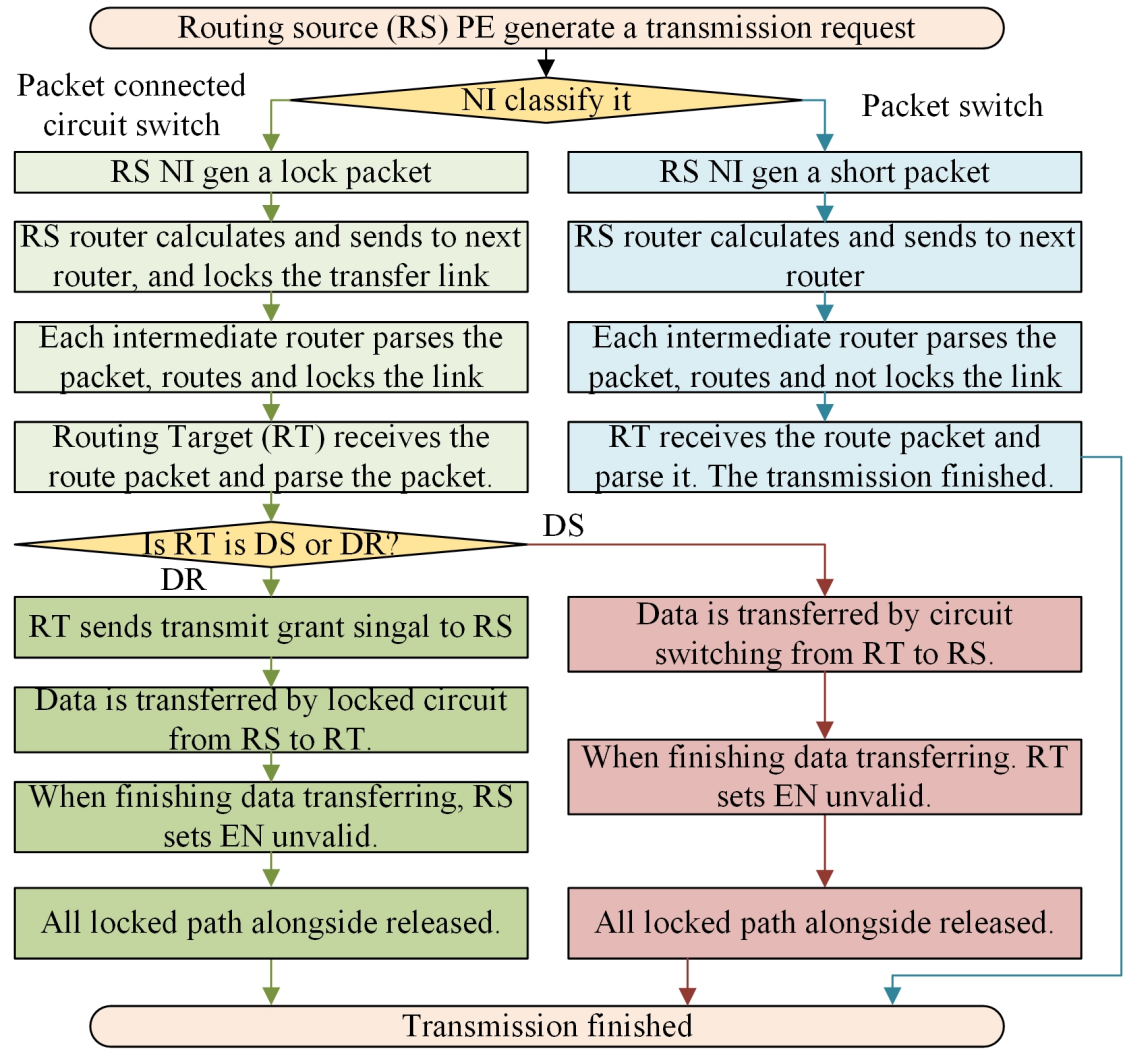
PaCHNOC routing flow. RS for routing source, RT for routing target, DS for data sender, and DR for data receiver.

Our comprehensive routing strategy and flow are depicted in Figure 6. The selection of the transmission method is determined automatically by the NoC system. In detail, the NI of the PaCHNOC system would dynamically choose its routing strategy based on factors of packet length in order to minimize average transmission latency. If the data size of the transmission task could fit in one flit, then packet switching mechanism would be used; else, the hybrid switching mechanism would be chosen.

The following abbreviations will be used afterwards:

1. **Routing source (RS)**: the node that initiates the transmission request;
2. **Routing target (RT)**: the node that accepts the transmission request;
3. **Data sender (DS)**: the node that sends data, could be RS or RT;
4. **Data receiver (DR)**: the node that receives data, could be RS or RT.

Figure 6 shows transmission flow for PaCHNOC. When a transmission generated by a node, firstly the NI module would classify the packet if it is a short packet or a long packet according to the packet size. Then, the NoC would use different switch strategy according to the classification. For PaCHNOC, there is a novel design that for a hybrid transmission routing source could be data sender or data receiver. For a long packet, whether RT is DS or DR would further influence the transmission process.

#### 3.3.1. Packet-Connected Circuit Mechanism

The research presents a proposed transmission task for NoC that consists of three distinct processes: circuit establishment and locking, data transmission, and circuit cancellation. These steps are illustrated in Figure 6.

**Establishing and Locking**: The network interface completes the preparation of source routing packets according to the requirements of the routing source. To establish a data transmission, the tasks for each router are as follows.

1. The router maintains the occupancy status of adjacent nodes.
2. According to the destination address of the route and the occupancy status of adjacent nodes, temporarily lock the in-port and out-port paths of the node according to data transfer direction, and send routing packets to the determined direction.
3. The routers along the transmitting path lock the circuit to establish a directional locked data path.
4. If Routing source (RS) is Data sender (DS), then Routing target (RT) node grants the transmission request. If RT is DS, RT could start data transmission directly.
5. The routers along the transmitting path keep path locked to support payload transmission on the circuit.

**Transmitting**: After the circuit is locked, the DS starts to transmit payload on the circuit to the data receiver (DR).

**Canceling**: When the transmission finishes, if RS is DS, RS sets unlock bit. All nodes along the constructed path are unlocked and release the circuit resources. If RT is DS, RT sets unlock bit.

**Figure 7:**
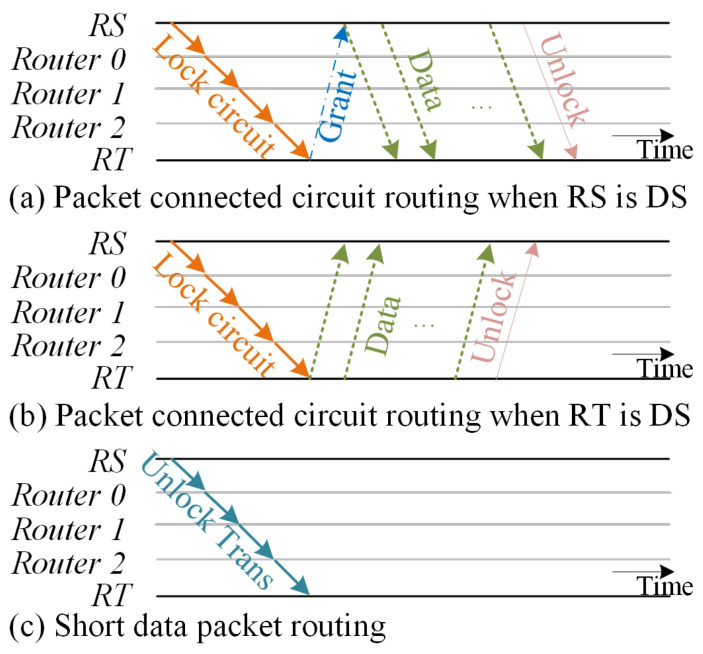
Mixed routing pipelines diagram. (**a**) packet-connected circuit routing pipelines when RS is DS; (**b**) packet-connected circuit routing pipelines when RS is DT; (**c**) short data packet routing pipelines.

Figure 7a,b shows how hybrid switching works. For an innovative and practical design, the routing source could be data sender whose transmission process is shown in Figure 7a; or the routing target could be data sender whose transfer flow is depicted in Figure 7b. There is circumstance when a module connects on the slave port of buses and it could ask data transmission from another module for MPSoC.

#### 3.3.2. Short Data Packet Routing Mechanism

The uncertainty in length of a data transaction is a notable aspect in the utilization of MPSoC. In the context of the base station system, many configuration vector packages and short data packets are commonly utilized. These include, such as, user data and control data of Physical Uplink Control Channel (PUCCH) and Physical Downlink Control Channel (PDCCH) [31].

In this case, the utilization of the temporary circuit locking technique results in a significant increase in network occupancy, leading to a decrease in overall performance. When employing short packet switching, data can be wrapped in one packet, the need for reordering is not necessary, resulting in a reduced silicon overhead. Additionally, the possibility of network occupancy is diminished. Each node employs a sending and forgetting method, which allows for efficient utilization of the routing resource without occupying it entirely. Figure 7c shows the pipeline for short data packet transmission which transfer the flit node by node without locking the circuit.

**Figure 8:**
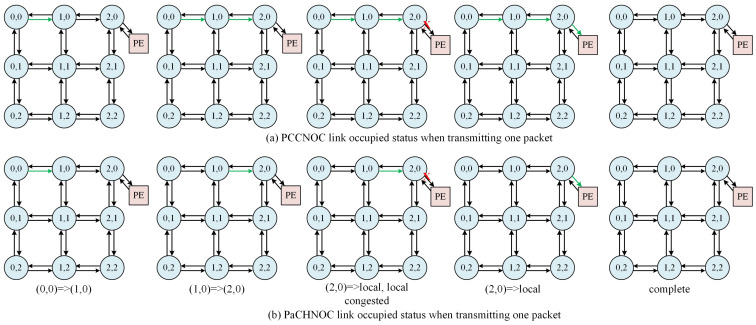
Link occupation status for PCCNOC and PaCHNOC when transmitting short packet. Green arrow is occupied link and red cross means congestion happens.

Figure 8 shows the difference for PCCNoC and PaCHNoC when a short data packet is transmitted from (0, 0) to (2, 0) where red X at (2, 0) means the path is occupied by other transactions which causes traffic congestion. Since PCCNOC adopts packet-connected circuit mechanism for short packet transmission, PCCNOC would lock circuits first which make the circuits occupied heavily. Instead, PaCHNOC uses packet switch strategy for short packet transmission which not only save transaction time but also reduce the system burden.

The features of streaming processing are relatively stable data flow, low latency and high bandwidth. The data transmitting on the NoC is composed of large volume of long task data and small volume of configuration data, yet the number of data transactions and the number of configuration vector transactions are on the same order. To better adapt to the application of stream processing on chip systems, PaCHNOC adopts a hybrid switching method, fully leveraging the ability of circuit switching to transmit long data; and packet switching to transmit short data to maximize the performance of stream processing on-chip systems.

## 4. The Proposed Architecture

The primary characteristic of PaCHNOC can be summarized as follows.

1. The PaCHNOC system is characterized by its buffer-less architecture, resulting in a minimal silicon cost.
2. To transfer long data, the PaCHNOC automatically classifies the data size, and transfers long data using packet-connected circuit. Thus, data buffer and its induced delay in each routing node is eliminated.
3. To transfer short data, the PaCHNOC also automatically classifies the data size, and the short data can therefore be carried by a single routing packet. An entire packet transmission is conducted by one routing packet, reorder buffer is thus not necessary.
4. The high throughput is achieved due to the efficient data transfer and the utilization of established circuits. High efficiency achieved by optimal performance with low silicon overhead.
5. The PaCHNOC system employs multiplexing circuits that support both circuit switching and packet switching to save connection cost.

In contrast to conventional NoC routers, the router for PaCHNOC does not have input and output buffers. The router and NI module of the proposed NoC have been improved from our early PCCNOC in order to facilitate the implementation of the mixed routing technique.

### 4.1. Router

**Figure 9:**
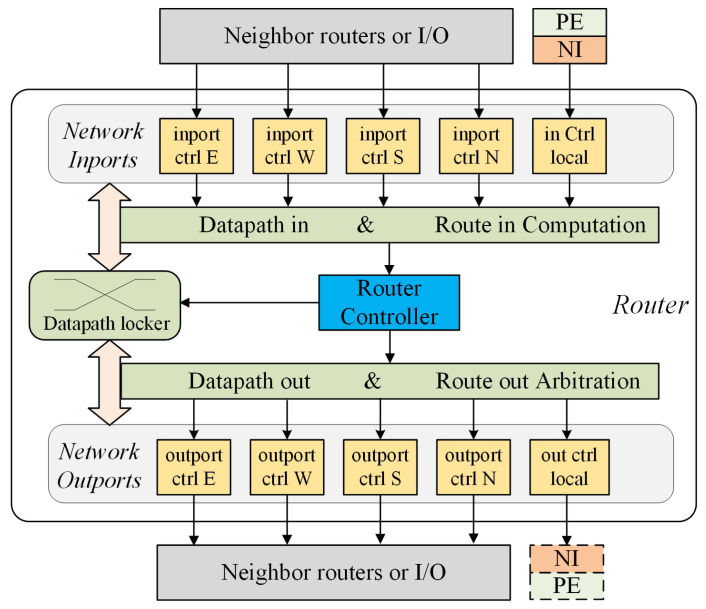
The proposed router architecture.

The router module serves as the essential component of the NoC system. The router is composed of a router controller (RoC), locking circuits (LC) logic, and network in and out ports, as depicted in Figure 9. The NI module in Figure 9 would be discussed in Section 4.2.

**Figure 10:**
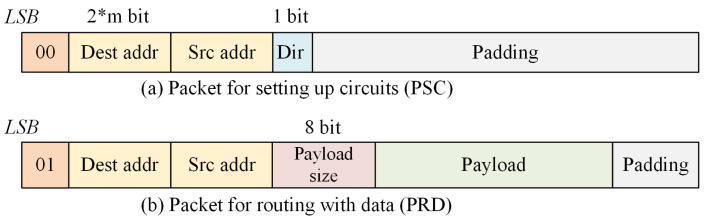
The format of the routing packets.

**Router controller (RoC)**: Responsible for routing information processing and maintaining. If the resource is available and routing is needed, the RoC will decide the destination of the incoming packet and the later circuit status. The decision whether to lock the circuit is made by the RoC according to packet content. If the packet for setting up circuits (described in Figure 10) is received, RoC would notify LC to lock the relative path. If packet for routing with data (depicted in Figure 10) is received, RoC would transfer the packet without locking circuits. If the link has been established, it will act as a transfer logic to send the data through the LC. If unlocking signal is requested, the RoC will send request to LC to release the locked circuits between input and output after data transmission. Various techniques exist for performing routing calculations, including the utilization of lookup tables or global settings [32]. To enhance the flexibility and to simplify the design of the NoC, this approach employs a distributed method for calculating routing nodes. Both XY and YX routing methods can be specified.

**Locking Circuit (LC)**: Composed of the router in computation logic, route out arbitration logic, and datapath locker. Router in computation logic would parse the packet and send the result to RoC. Router Arbitration logic records and keeps updating the occupancy status of adjacent nodes. The routing decision is sent to the datapath locker by RoC to implement output selection and the relative paths are locked right after routing. Those paths become parts of the circuits to be established. After receiving the unlock signal, LC would unlock the relative paths to release valid path resources.

**Network in and out ports**: Includes module entrance configuration circuit and output configuration circuit for linking a PE to a router and connecting neighbor routers and IOs.

**Figure 11:**
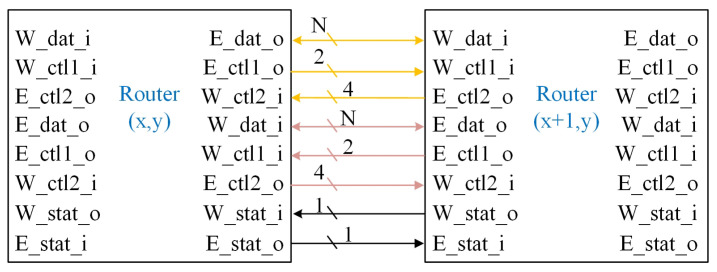
Interface signals for routers.

To trace the state information of surrounding routing nodes, PaCHNOC uses a 1-bit state signal as back-pressure information. The method used for virtual cut-through requires a multi bit credit to record the status of surrounding routing nodes [33]. By contrast, PaCHNOC only requires 1 bit of state information, which greatly reduces the complexity of the interface design. The interfaces of the routers are shown in Figure 11.

The interface is composed of two groups of ports. Each group contains a bandwidth-configurable (N is configurable) data port and six control ports. The detailed information for each part of interface is listed in Table 2. Ports include dat for transmitting data, ctl1 accompanying dat to send control signals, and ctl2 in reverse direction with dat for receiving exceptions, warnings, and other information. Stat is used to transmit the congestion status of adjacent nodes, in order to facilitate the calculation of routing direction by the current router.

CIMA [24] adopts a separate control path to transmit information and to lock the circuit, and its packet switching part using virtual cut-through method. In contrast, PaCHNOC uses back-pressure to control packet switching on the NoC, and it utilizes the transmission circuit for both control and data packets.

There are two different types of packets distinguished by the first two bits at LSB, as shown in Figure 10. (1) Packet for setting up circuits (PSC), which is used to establish a long data transmission path. Dir is used to indicate whether RS is DS or DR. (2) Packet for routing with data (PRD), which is used for short-size data transmission by packet switching without locking the transmission path on the way. RoC distinguishes the packet type by the leading two bits.

### 4.2. Network Interface

**Figure 12:**
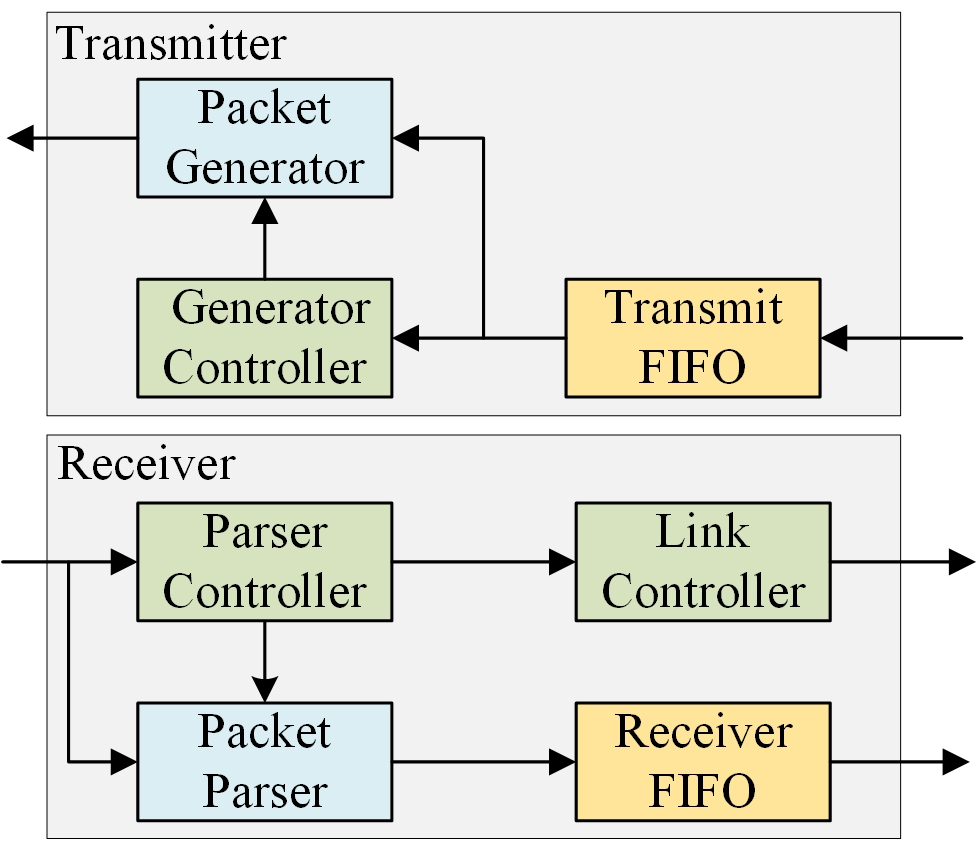
Network interface architecture.

The NI module is used for packing and uploading data from the local module. It also works for unpacking and downloading data to the local module. It comprises two components, namely a transmitter and a receiver. The transmitter comprises a packet generator and a generator controller (as depicted in Figure 12), which are employed to regulate the production of various categories of data packets in response to specific demands. The packet generator partitions and encapsulates the package according to the generator controller’s decision. The receiver incorporates a parser controller that serves the purpose of extracting control data, a link controller generating control information, and a packet parser to disassemble payload for the target. Subsequently, it transmits the payload to the local module. When handling burst traffic (which is a long data packet), the NI module will send a back-pressure signal to the PE not allowing it to transmit data until the circuit is locked.

Both the transmitter and receiver are equipped with First-In-First-Out (FIFO) buffers, which serve the purpose of synchronization within the context of a Global Asynchronous Local Synchronous (GALS) system. In physical design, the logical transmission FIFO and the logical receive FIFO is actually one physical device.

### 4.3. Pipeline

The fundamental packet switching NoC requires the implementation of lengthy pipelines within routers [29], as shown in Figure 4a. This is due to the inclusion of buffers and virtual channels. The processes of writing and reading data from buffers are time-consuming. In a general sense, the process of packet switching typically involves the execution of seven distinct phases [29]. The following components are included: Buffer Write (BW), Route Compute (RC), Virtual Channel Allocation (VA), Switch Allocation (SA), Buffer Read (BR), Switch Traversal (ST), and Link Traversal (LT).

The proposed solution requires the utilization of only two pipelines. Our PaCHNOC, being a buffer-less NoC, effectively minimizes memory access latency. The integration of RC and SA, ST, and LT results in a total of two clock cycles for each node for packet switching (shown in Figure 4b). Conversely, for the locked circuit, the data transmission circuit of each node can be set as a combination circuit or a pipeline register. Along the locked data path, most nodes are set as combination logic, while few intermediate nodes are set to pipeline register according to the length of the whole path.

The routing flow and pipeline are depicted in Figure 6 and Figure 7, respectively.

## 5. Implementation and Evaluation

### 5.1. Simulation Setup

Noxim [34] simulator is used to model and simulate the traditional packet switching NOC. Noxim is a well-known cycle accurate NoC simulator to analyze performance of a NoC. In order to justify the performance of the proposed work, routers and network interfaces for PaCHNOC and other related works have been implemented in Noxim.

To evaluate the performance of different NoCs, a baseline, VCS [35], CIMA [24], and PCCNOC [6] are used for experiment. The parameters of these NoCs are mentioned in Table 3.

VCS [35] (virtual circuit switch) uses a flow-control policy that exclusively reserves a virtual path for routing packets in an adaptive mode for each communication flow in order to guarantee that delivery of all packets belongs to the same communication flow. It allows sharing of the reserved path among packets of different communication flows. The method needs additional memory unit for storing all unordered packets, and needs extra channels for path reservation.

A CIMA [24] router consists of two related networks: a data network for sending and receiving packets (e.g., requests and responses) and a control network for setting up circuits. Request packets are routed using VCT switching. When a request packet arriving to the destination, it is queued within the Last-Level Cache (LLC) and waits for its lookup time. If the tag lookup indicates a hit, LLC controller will notify the NI. The NI creates a control packet. On receiving a control packet in a router, the packet is passed through a route computation unit and circuit reservation logic in parallel.

PCCNOC [6] is our previous-generation work that establishes circuits through packet switching and then transmits data through circuits. It is characterized by making full use of the flexibility of packet-switched networks and the reliability of circuit switching, and has obvious performance advantages in the case of large data transmission. However, when there is a large amount of short data packets transmitting in the service, the advantages of PCCNOC cannot be fully realized, and even the performance will degrade due to the continuous locking of the circuit.

### 5.2. Simulation Result

#### 5.2.1. Latency and Throughput

**Figure 13:**
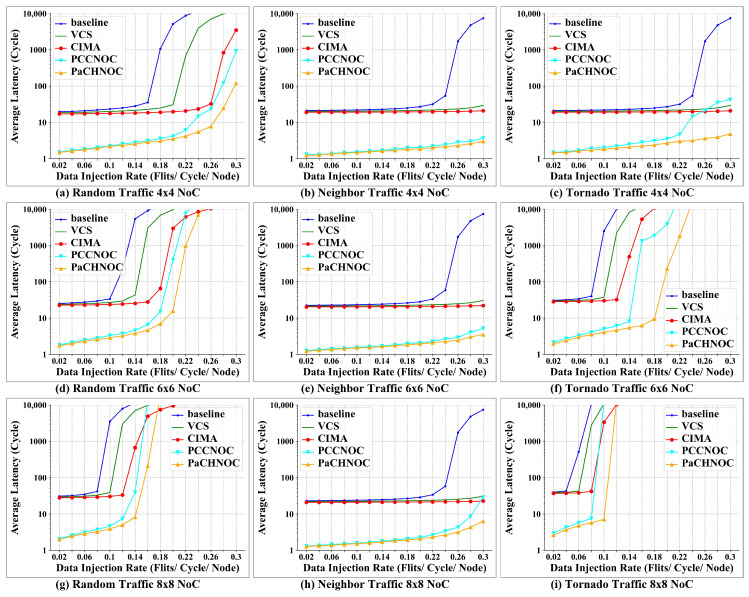
Average latency simulation results under (**a**) uniform random traffic for 4×4 NoC mesh, (**b**) neighbor traffic for 4×4 NoC mesh, (**c**) tornado traffic for 4×4 NoC mesh, (**d**) uniform random traffic for 6×6 NoC mesh, (**e**) neighbor traffic for 6×6 NoC mesh, (**f**) tornado traffic for 6×6 NoC mesh, (**g**) uniform random traffic for 8×8 NoC mesh, (**h**) neighbor traffic for 8×8 NoC mesh, and (**i**) tornado traffic for 8×8 NoC mesh.

Simple traffic models, such as the uniform random model, are useful for NoC designers in acquiring insights by stressing the network with a regular predictable traffic pattern. However, utilizing traffic models that mimic realistic traffic behavior, such as neighbor and tornado rule, is significantly important [36]. Our experiments were designed to trace NoC behavior under different traffic models with an 4×4, 6×6, 8×8 NoC mesh using Noxim, and the execution time was 100,000 cycles. The NoCs are motivated by random, neighbor, and tornado traffic model. Firstly, we evaluate PaCHNOC with baseline, VCS [35], CIMA [24], and PCCNOC under random, neighbor, and tornado traffic, respectively. Figure 13a–c, Figure 13d–f, and Figure 13g–i depict the average latency according to the different injection rate in 4×4, 6×6, and 8×8 NoC meshes, respectively. It can be seen that the saturation point of injection rate for PaCHNOC is higher than that of all other related work, and the average latency is lower at injection rates from 0.02 to 0.03 for the all three different dimensions. For the injection rate at 0.1, the average latency reduced by PaCHNOC is 92%, 91%, 90%, and 5% for the 4×4 mesh, 91%, 90%, 89%, and 15% for the 6×6 mesh, and 96%, 94%, 93%, and 19% for the 8×8 mesh in comparison with baseline, VCS [35], CIMA [24] and PCCNOC, respectively. For the injection rate at 0.1, PaCHNOC reduced average latency by 93%, 90%, 88% and 12% under random traffic model, 94%, 93%, 83% and 4% under neighbor traffic model, 98%, 95%, 92% and 21% under tornado traffic model compared with baseline, VCS, CIMA, and PCCNOC, respectively.

Secondly, we focus our attention to real benchmark traffic. Snipersim 6.1 [37] was used to trace real benchmark traffic, and the router and network interface of VCS [35], CIMA [24], PCCNOC [6], and PaCHNOC were implemented on Snipersim. We selected five PARSEC [38] benchmark applications (viz. fluidanimate, vips, X264, blackscholes, and dedup) due to their variability in offering both communication and computation-intensive workload. The simulation was executed on an 8×8 NoC mesh and the execution time was 100,000 cycles.

**Figure 14:**
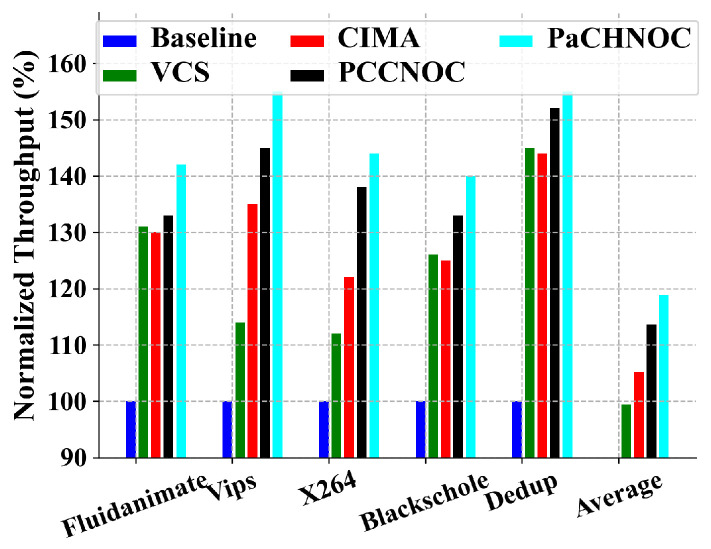
Throughput (in Mbps) normalized to baseline on 8×8 mesh under the PARSEC benchmarka suite.

In the case of PARSEC benchmark application, the maximum gain in terms of average throughput achieved by four techniques is observed as 25%, 31%, 40%, and 47% for VCS [35], CIMA [24], PCCNOC [6], and PaCHNOC, respectively, compared with the baseline (see Figure 14).

**Figure 15:**
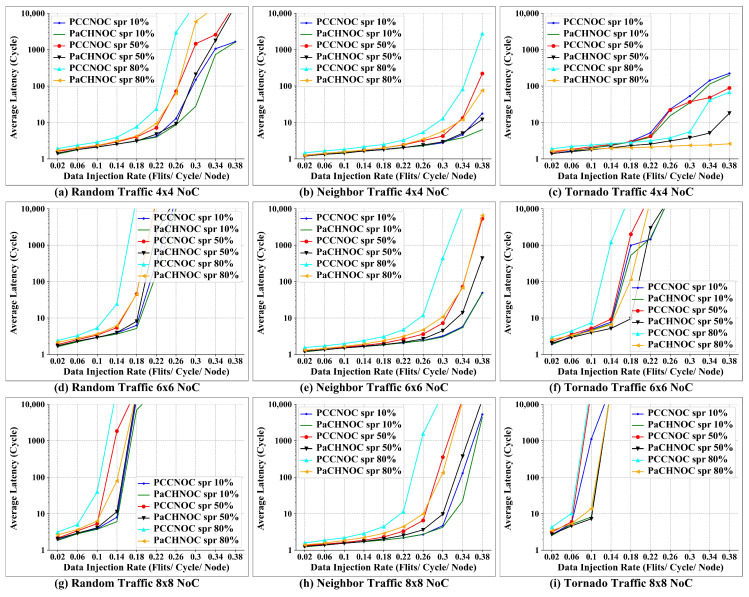
Average latency comparison between PaCHNOC and PCCNOC with different SPR under (**a**) uniform random traffic for 4×4 NoC mesh, (**b**) neighbor traffic for 4×4 NoC mesh, (**c**) tornado traffic for 4×4 NoC mesh, (**d**) uniform random traffic for 6×6 NoC mesh, (**e**) neighbor traffic for 6×6 NoC mesh, (**f**) tornado traffic for 6×6 NoC mesh, (**g**) uniform random traffic for 8×8 NoC mesh, (**h**) neighbor traffic for 8×8 NoC mesh, and (**i**) tornado traffic for 8×8 NoC mesh. Spr stands for short packet rate.

Thirdly, let us evaluate the improvement for PaCHNOC with our previous work on PCCNOC. As aforementioned, PaCHNOC boost switching abilities when there are considerable small size packets, so experiments are designed to evaluate the performance under different short packet rates (SPR). Short packet stands for one packet can carry the complete data. We used three traffic models: random, neighbor, and tornado, with an SPR of 10%, 50%, and 80%, and NOC dimensions are 4×4, 6×6, and 8×8, respectively. Figure 15a–c, Figure 15d–f, and Figure 15g–i depict the average latency according to the different injection rate in the 4×4, 6×6, and 8×8 NoC mesh, respectively.

As can be seen from the results, the higher the SPR is, the longer the average latency is. The average latency of PaCHNOC is lower than that of PCCNOC under the same SPR. PaCHNOC reduces overall latency by 17%, 22%, and 47% for the 4×4 mesh, 9%, 33%, and 49% for the 6×6 mesh, 7%, 32%, and 53% for the 8×8 mesh, for SPR 10%, 50%, and 80%, respectively, compared with PCCNOC. Furthermore, the overall latency reduced by PaCHNOC is 9%, 23%, and 45% under the random traffic model, 18%, 63%, and 67% under the neighbor traffic model, 6%, 35%, and 55% under the tornado traffic model, for SPR 10%, 50%, and 80%, respectively, in contrast with PCCNOC.

**Figure 16:**
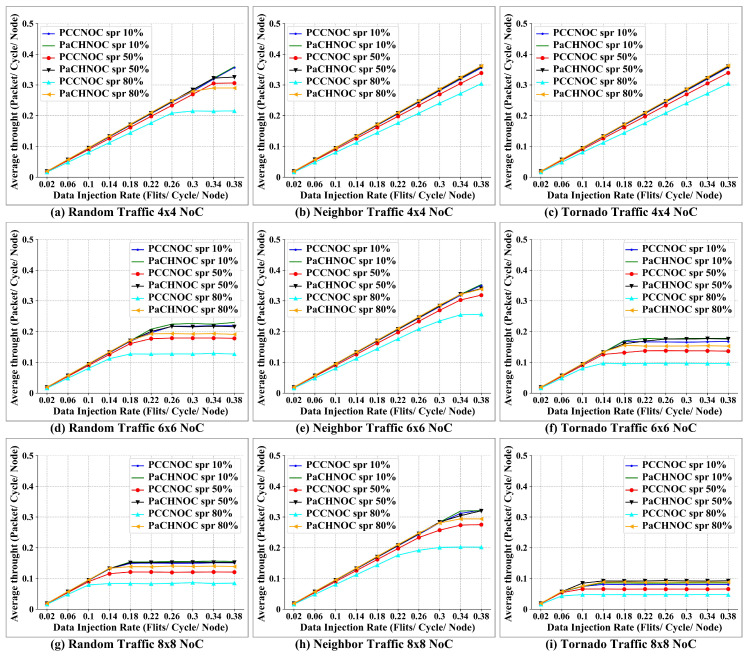
Throughput comparison between PaCHNOC and PCCNOC with different SPR under (**a**) uniform random traffic for 4×4 NoC mesh, (**b**) neighbor traffic for 4×4 NoC mesh, (**c**) tornado traffic for 4×4 NoC mesh, (**d**) uniform random traffic for 6×6 NoC mesh, (**e**) neighbor traffic for 6×6 NoC mesh, (**f**) tornado traffic for 6×6 NoC mesh, (**g**) uniform random traffic for 8×8 NoC mesh, (**h**) neighbor traffic for 8×8 NoC mesh, and (**i**) tornado traffic for mesh 8×8 NoC. Spr stands for short packet rate.

Figure 16a–c, Figure 16d–f, and Figure 16g–i demonstrate the average throughput according to the different injection rate in 4×4, 6×6, and 8×8 NoC mesh, respectively. As we can see from the result, the average throughput improves as the SPR increases, then stays stable after reaching the saturation point. For the same SPR, PaCHNOC achieves a higher network throughput the PCCNOC. In summary, PaCHNOC improve overall throughput by 1%, 6%, and 21% for 4×4 mesh, 3%, 13%, and 35% for 6×6 mesh, 2%, 17%, and 46% for 8×8 mesh, for SPR 10%, 50%, and 80%, respectively, compared with PCCNOC. Besides, the overall throughput improved by PaCHNOC is 2%, 12%, and 37% under the random traffic model, 1%, 7%, and 24% under the neighbor traffic model, 3%, 16%, and 37% under the tornado traffic model, for SPR 10%, 50%, and 80%, respectively, in contrast with PCCNOC.

When the SPR increases, PCCNOC needs to constantly spend time locking the circuit, transmitting data, and releasing the circuit. In conclusion, the time spent on transmitting effective data is much less in contrast with the redundant time spent on locking and releasing circuits, resulting in a loss of system performance. PaCHNOC solves this problem by providing adaptive routing strategy and implementing a packet switching for short packets.

#### 5.2.2. Saturation Point

**Figure 17:**
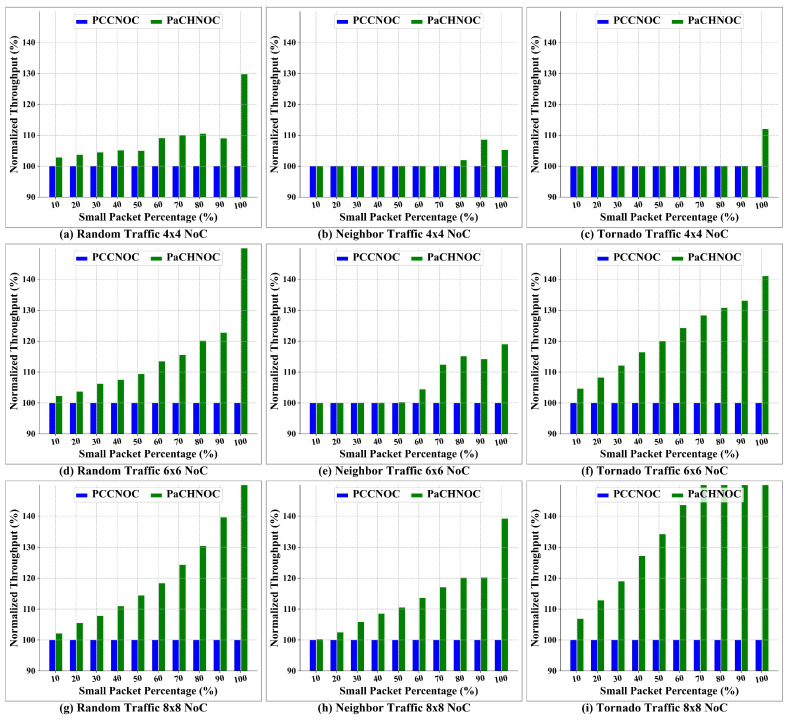
Saturation point comparison between PaCHNOC and PCCNOC with different short packet percentages under (**a**) uniform random traffic for 4×4 NoC, (**b**) neighbor traffic for 4×4 NoC, (**c**) tornado traffic for 4×4 NoC, (**d**) uniform random traffic for 6×6 NoC, (**e**) neighbor traffic for 6×6 NoC, (**f**) tornado traffic for 6×6 NoC, (**g**) uniform random traffic for 8×8 NoC, (**h**) neighbor traffic for 8×8 NoC, and (**i**) tornado traffic for 8×8 NoC.

Next, we compared NoC saturation points with different SPR, and the results are normalized to PCCNOC. Three traffic models are adopted, i.e., random, neighbor, and tornado, and the dimensions of NOC are 4×4, 6×6, and 8×8, respectively. The results are shown in Figure 17a–c, Figure 17d–f, and Figure 17g–i for 4×4, 6×6, and 8×8 NoC meshes with different short packet percentages. It can be seen from the results that for the same dimension, the saturation point of PaCHNOC is higher (the higher the better) than that of PCCNOC under the same traffic. In short, the saturation point of 4×4 NoC is increased by 103%, by 112% for 6×6 NoC, and by 119% for 8×8 NoC when the SPR is 10%. In addition, the saturation point under the random traffic model is increased by 113%, by 106% under the neighbor traffic model, and 119% under the tornado traffic model when the SPR is 10%.

### 5.3. Application Traffic

**Figure 18:**
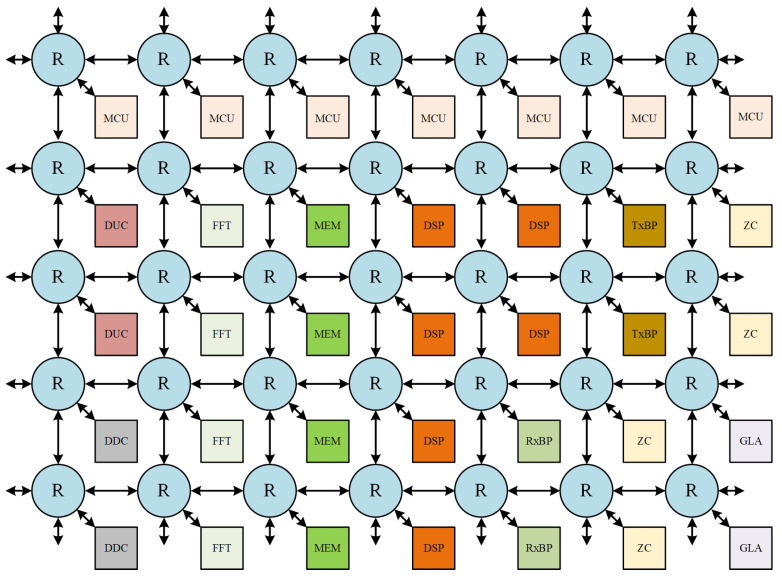
A base station system using PaCHNOC. GLA is the Galois processor. ZC is the Zaddoff Chu module. RxBP is the receiver bit field accelerator cluster. TxBP is the transmitter bit field accelerator cluster. FFT stands for Fast Fourier Transform. DUC is the digital up-conversion. DDC is short for digital downconversion.

In this section, we will use our research PaCHNOC to integrate the baseband subsystem of a small base station for 5G, as a real-time parallel data streams processing scenario. The features are: (1) the MCU is responsible for generating tasks or task chains; (2) each task within the task chain is associated with a task configuration package; (3) the functional modules and storage modules are configured for task execution using the short packet exchange protocol; (4) a circuit locked packet switching protocol is utilized to secure a specific storage and functional module, initiating data transmission; (5) following the completion of data transmission, the task job is executed promptly, and the task execution status is reported; and (6) the locked transmission circuit is immediately unlocked upon task execution completion, and the unlocking status is reported simultaneously. Figure 18 shows an example for a base station system using PaCHNOC.

**Figure 19:**
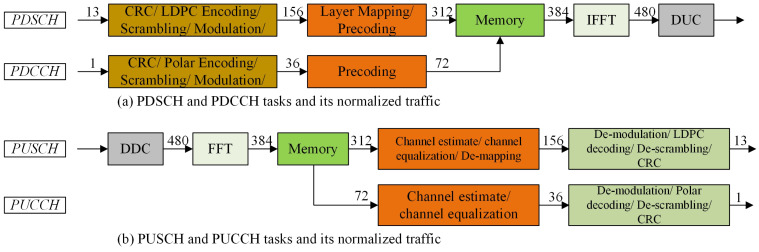
Task flows and their normalized traffic for the NR system.

We use real applications to evaluate NOC performance by the NoC model, as shown in Figure 18. We analyze the sending and receiving process of one slot of the 5G NR system. The tasks of Physical Downlink Shared Channel (PDSCH), PDCCH, Physical Uplink Shared Channel (PUSCH), PUCCH, and their corresponding task streams, along with their normalized traffic, is depicted in Figure 19 [39]. In addition to the data traffic, each task represented in Figure 19 needs to be configured by the MCU, and the sizes of task configuration data are relatively small.

**Figure 20:**
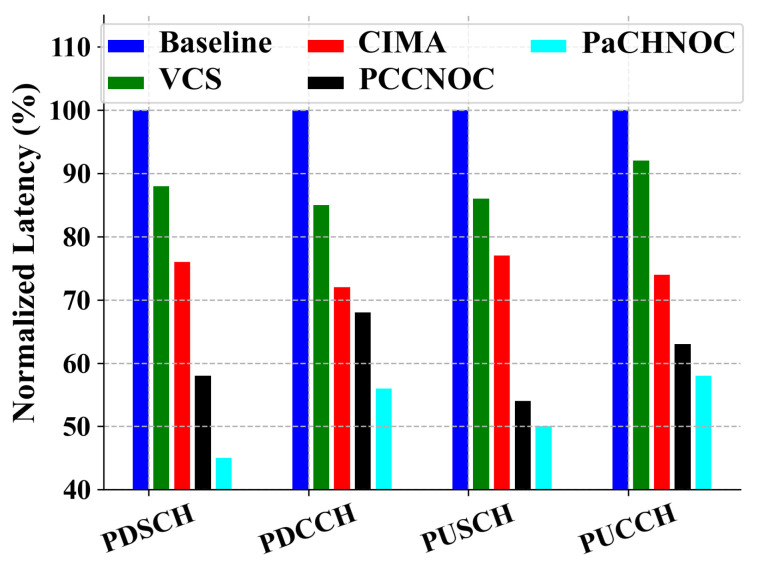
Average latency for different NoC model normalized to baseline on 7×5 NoC mesh under NR real application tasks.

We used Noxim to build the 7×5 NOC architecture shown in Figure 18. A baseline VCS, CIMA, and PCCNOC are set as reference. The traffic model is designed by input and output data flow for tasks. The total propagation delay in the NOC is analyzed and the normalized latency is given in Figure 20. It can be seen that the performance of PaCHNOC has 47%, 40%, 30%, and 14% gains compared with baseline, VCS, CIMA, and PCCNOC, respectively. Compared with PCCNOC, the gain of PaCHNOC mainly comes from the gain of configuration packet switching. Since the configuration packets are generally short packet, PaCHNOC does not need to lock the link. Therefore, the probability of being blocked when the link is established is smaller.

### 5.4. Synthesis Result

A router (including its NI) of PaCHNOC was achieved. Each router was synthesized with Synopsys Design Compiler using the TSMC 12 nm standard cell to extract the timing and area information. The clock frequency was 3 GHz. For the real application scenario, the bandwidth was set to 262 bits (256 bits data and 6 bits for control). Table 4 provides the synthesis results. It is worth mentioning that the area is the logic circuit overhead, excluding the overhead metal connection wire.

In order to compare with different methods, we configured our interface bitwidth to 48 bits (42 bits data and 6 bits for control). Due to the different processes used in different studies, we refer to the method in [40] to normalize the silicon area to 65 nm for comparison. PaCHNOC is compared with three packet switching NoCs, i.e., MRBS [20], MRCN [21], and SmartFork [41], and three hybrid NoCs, i.e., CIMA [24], VCS [35], and PCCNOC, as per our previous work [6]. Our proposal resulted in a decrease in area of 75.4% and 56.5%, respectively, in comparison with that of CIMA [24] and VCS [35]. This is because we used the same interface to handle packet switching and circuit switching, and only increased the additional bandwidth of the six control bits. Compared with PS NoC, the area used by our proposal is reduced by 90.1%, 89.9%, and 72.6% compared to that of MRBS [20], MRCN [21], and SmartFork [41], respectively. Compared with them, PaCHNOC saves the storage overhead of the re-order buffer and virtual channel. In contrast with our previous work PCCNOC, the area increases 2% but the performance improves 53% according to aforementioned discussion. PaCHNOC adds specific logic for short packet arbitration and handling, which resulted in a slight increase in area, yet improvement of the overall performance. The results of different studies are detailed in Table 5.

## 6. Conclusions

For high-performance, real-time parallel stream processing systems, we investigated special needs and designed our hybrid NoC based on both packet transmission and packet-connected circuit transmission. Based on our early significant work PCCNOC, we further improved the NoC performance by applying adaptive transmission mechanism. The improvement mainly comes from the new design PaCHNOC. It identifies and handles long data packets and short data packets separately. The long data packets are still transmitted through temporary circuits established and locked by routing. The short data packet is attached to the routing packet, and the transmission is completed while the route is established. Therefore, for short data packets, the relatively long route setup and locking time overhead of our early PCCNOC is eliminated. PaCHNOC achieved both short data transfer latency for the long data transmission, and low routing overhead cost for the short data transmission. This is a very important improvement for real-time systems with stringent time scheduling requirement for multiple parallel task issues, execution, and managements.

The improvements are summarized: (1) much lower routing overhead latency, i.e., 53%, compared with PCCNOC; (2) much higher transmission throughput, i.e., 46%, in comparison with PCCNOC; (3) higher saturation point, i.e., 19%, compared with PCCNOC; and (4) only a 2% silicon area increase compared with PCCNOC, and lower silicon cost of 75% in comparison with related hybrid NoC CIMA.

## Figures and Tables

**Table 1:** Parameter description for network latency calculation.

Abbreviations	Description
$T_{n}$	Network delay
$t_{r}$	Router pipeline delay
$t_{w}$	Wire delay per hop
*H*	Number of hops
$T_{c}$	Contention delay
$T_{s}$	Serialization delay (for multi-flit packets)
$t_{c}$	Time for transmitting a packet from source to destination using circuit switching.
*F*	Flits number
$t_{oa}$	Average occupied links when congestion happens, which is in range {1, H}.
$T_{hA}$	Average latency per node for hybrid NoC
$T_{pA}$	Average latency per node for packet switched NoC
$N_{hO}$	Total occupied link number for hybrid NoC
$N_{pO}$	Total occupied link number for packet switched NoC

**Table 2:** Detailed information for each direction at each port of a router.

Port Name	Bit Width	Direction	Function
dat	N bits	Double sided	Packet
ctl1_i	2 bit	DS to DR	To indicate whether the transmission is valid; To unlock the constructed path.
ctl2_o	4 bit	DR to DS	To notice the transaction has time violation; To state the data transmission fails; To authorize data transaction of data sender; To refuse data transmission.

**Table 3:** Comparing different NoC solutions.

NoC Modle	Features
Baseline	Mesh Router: 5 ports, 3 VCs/port, 5 flits/VC 2-stage speculative pipeline. Link: 1 cycle
VCS [35]	Data network: 5 ports/router, 4 VCs/port, 8 flits/VC 2-stage speculative pipeline, 32 bits flits width
CIMA [24]	Data network: 5 ports/router, 3 VCs/port Packet-switching mode: 2-stage speculative pipeline. Link: 1 cycle Circuit-switching mode: Bypassing pipeline stages, Link: 1 cycle Control network: 5 ports/router, NO VCs, 1-stage pipeline. Link: 1 cycle
PCCNOC [6]	Data network: 5 ports/router, no VCs/port Packet-switching mode: 2-stage speculative pipeline. Link: 0 cycle Circuit-switching mode: Bypassing pipeline stages, Link: total 2 cycle Control network: no dedicated control network
PaCHNOC	Data network: 5 ports/router, no VCs/port Packet-switching mode: 2-stage speculative pipeline. Link: 0 cycle Circuit-switching mode: Bypassing pipeline stages, Link: total 2 cycle Control network: no dedicated control network

**Table 4:** PaCHNoC router synthesis result.

	Bandwidth (Bits)	Frequency (GHz)	Area (μm^2^)	Combinational Area (μm^2^)	Noncombinational Area (μm^2^)	Power (mW)
PaCHNoC	263	3	3180.81	1406.78	1774.03	3.2

**Table 5:** Synthesis result comparison with other studies.

Research	Bandwidth (Bits)	Technology (nm)	Frequency (Ghz)	Area (μm^2^)	Normalized Area (μm^2^) ^1^	Power (mW)
MRBS [20]	34	SAED 32	0.5	87,753	362,066	11.8
MRCN [21]	34	SAED 32	-	85,616	353,249	8.5
CIMA [24]	128	32	2	35,000	144,409	-
SmartFork [41]	-	45	1	62,370	130,130	5.58
VCS [35]	-	TSMC 45	2	46,000	95,975	17.1
PCCNOC [6]	49	TSMC 12	3	1189	34,885	1.4
Our work	48	TSMC 12	3	1213	35,590	1.4

^1^ $\mathrm{Normalized}\_\mathrm{area}=\mathrm{area}\;\times {\left(\frac{65\;\mathrm{nm}}{\mathrm{process}}\right)}^{2}$.

## Data Availability

The data that support the findings of this study are available on request from the corresponding author.
